# Design and 3D printing of variant pediatric heart models for training based on a single patient scan

**DOI:** 10.1186/s41205-021-00116-6

**Published:** 2021-08-31

**Authors:** Carina Hopfner, Andre Jakob, Anja Tengler, Maximilian Grab, Nikolaus Thierfelder, Barbara Brunner, Alisa Thierij, Nikolaus A. Haas

**Affiliations:** 1 Department of Pediatric Cardiology and Pediatric Intensive Care, LMU Klinikum, Campus Großhadern, Marchioninistr. 15, 81377 Munich, Germany; 2 Department of Cardiac Surgery, LMU Klinikum, Campus Großhadern, Marchioninistr. 15, 81377 Munich, Germany

**Keywords:** 3D Printing in Medicine, Non-patient-specific 3D Modeling, Hands-on Training, Pediatric Cardiology, 3D Heart Models, Pulsatile Heart Model

## Abstract

**Background:**

3D printed models of pediatric hearts with congenital heart disease have been proven helpful in simulation training of diagnostic and interventional catheterization. However, anatomically accurate 3D printed models are traditionally based on real scans of clinical patients requiring specific imaging techniques, i.e., CT or MRI. In small children both imaging technologies are rare as minimization of radiation and sedation is key. 3D sonography does not (yet) allow adequate imaging of the entire heart for 3D printing. Therefore, an alternative solution to create variant 3D printed heart models for teaching and hands-on training has been established.

**Methods:**

In this study different methods utilizing image processing and computer aided design software have been established to overcome this shortage and to allow unlimited variations of 3D heart models based on single patient scans. Patient-specific models based on a CT or MRI image stack were digitally modified to alter the original shape and structure of the heart. Thereby, 3D hearts showing various pathologies were created. Training models were adapted to training level and aims of hands-on workshops, particularly for interventional cardiology.

**Results:**

By changing the shape and structure of the original anatomy, various training models were created of which four examples are presented in this paper: 1. Design of perimembranous and muscular ventricular septal defect on a heart model with patent ductus arteriosus, 2. Series of heart models with atrial septal defect showing the long-term hemodynamic effect of the congenital heart defect on the right atrial and ventricular wall, 3. Implementation of simplified heart valves and addition of the myocardium to a right heart model with pulmonary valve stenosis, 4. Integration of a constructed 3D model of the aortic valve into a pulsatile left heart model with coarctation of the aorta. All presented models have been successfully utilized and evaluated in teaching or hands-on training courses.

**Conclusions:**

It has been demonstrated that non-patient-specific anatomical variants can be created by modifying existing patient-specific 3D heart models. This way, a range of pathologies can be modeled based on a single CT or MRI dataset. Benefits of designed 3D models for education and training purposes have been successfully applied in pediatric cardiology but can potentially be transferred to simulation training in other medical fields as well.

## Background

Simulation training is an inherent part in professions associated with high-risk and considerable demands regarding knowledge and skills. A well-known example is a flight simulator used for education and training in aviation [1]. This concept has been transferred to the medical field and has become the gold standard for resuscitation training. Basic courses are recommended for the general public, i.e., also non-physicians, and mandatory for medical personnel. Advanced courses are intended specifically for medical professionals involved in resuscitation teams [2]. There are other medical fields adopting the concept of simulation in education and training and, thereby, making use of 3D printed anatomical models. Applications of 3D printed models in training courses have been described amongst others for neurosurgery, otolaryngology, cardiac surgery and adult cardiology [1, 3–6]. The standard methodology for education in medicine, also in pediatric cardiology, however, still follows the apprenticeship-model, i.e. the trainee observes, takes over step-by-step and practices under supervision until he/she can perform an intervention independently [7]. The effectiveness of simulator training in medicine was proven and it was shown that the request and need for 3D printed models in medical education increases continuously [1, 8].

3D printed heart models are commonly generated from computed tomography (CT) and magnetic resonance imaging (MRI) scans of patients. These imaging techniques involve harmful effects such as radiation for CT and often sedation for MRI in young children. Therefore, the acquisition of CT and MRI scans of patients with congenital heart disease (CHD) is not always indicated. The ALARA (“as low as reasonably achievable”) principle [9] as well as the Image Gently Alliance for pediatric patients [10] make sure that radiation doses are low for CT imaging. For MRI imaging, specific techniques have been reported for infants and small children which avoid sedation [11] or reduce sedation time by improving MRI protocols in terms of speed [12]. However, ultrasound imaging is the imaging technique of choice for pediatric patients, if CT or MRI imaging do not provide additional information [13]. For 3D modeling and 3D printing this results in a shortage of CT and MRI scans for specific pathologies, as 3D ultrasound images are not (yet) suitable to generate 3D models of the entire heart.

The aim of this study is to demonstrate the possibility of creating variant 3D printed CHD models for educational and training purposes by modifying existing patient-specific 3D heart models instead of starting with and relying on CT or MRI data.

## General Methods in 3D Modelling

Digital engineering techniques allow the modification of computational meshes and, thus, the creation of variant heart models based on a single patient scan. Some of the tools and workflows for changing the anatomy of 3D heart models are presented in this study. The software suite that was used (Materialise Mimics Innovation Suite MIS 22.0, Materialise NV, Leuven, Belgium) offers the possibility to segment 2D image stacks and convert them into STLs, i.e., 3D virtual surface models (Materialise Mimics 22.0). On the other hand, it provides a variety of tools allowing engineering operations to modify the structure of surface meshes (Materialise 3-matic 14.0).

Patient-specific 3D heart models are created by segmentation of the contrast-enhanced blood volume visible in a CT or MRI image stack. Basic design operations are then applied to the generated 3D model, which do not change the anatomy but the surface quality of the digital models (e.g., smoothing). This process has been comprehensively described in the literature, e.g., by Grab et al. [14]. Heart models are usually hollowed by adding a shell around the blood volume to the outside in a certain user-defined distance and vessel endings are cut open to access the intracardial structures. In this report, we want to focus on the new approach of creating 3D heart variants based on these patient-specific versions. Digital engineering workflows for global or local scaling, creating windows or anatomical defects, simulation of vessel pathways and integration of muscular heart structures are presented in the following.

### Scaling

**Fig. 1:**
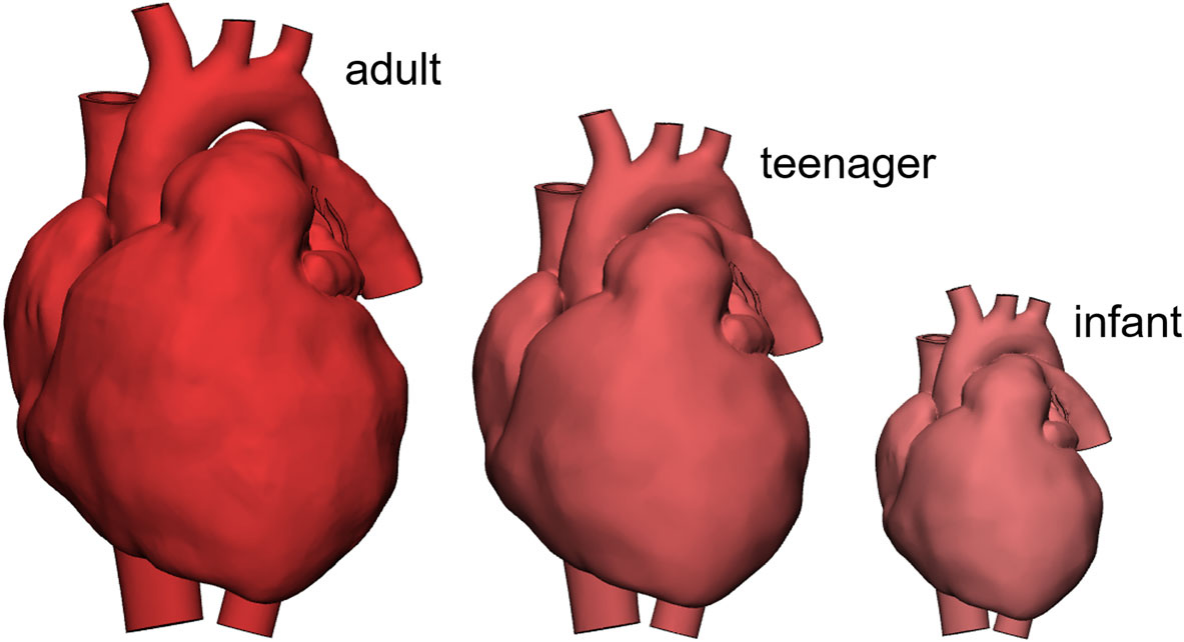
3D heart models of different sizes based on a single patient dataset. The adult sized heart was created from a CT scan. It was then scaled by a multiplying factor of 0.8 for the teenage heart and by 0.55 for the infant heart

The simplest engineering operation applicable to 3D models is scaling. The size of the original model can be changed by a scaling factor or by assigning delta values in the three dimensions x, y, and z. To maintain the proportions of the heart, uniform scaling in all dimensions is recommended. Scaling is an effective tool to create heart models of different size representing patient ages from infant to adult based on a single CT or MRI scan (Fig. 1).

### Openings and windows

**Fig. 2:**
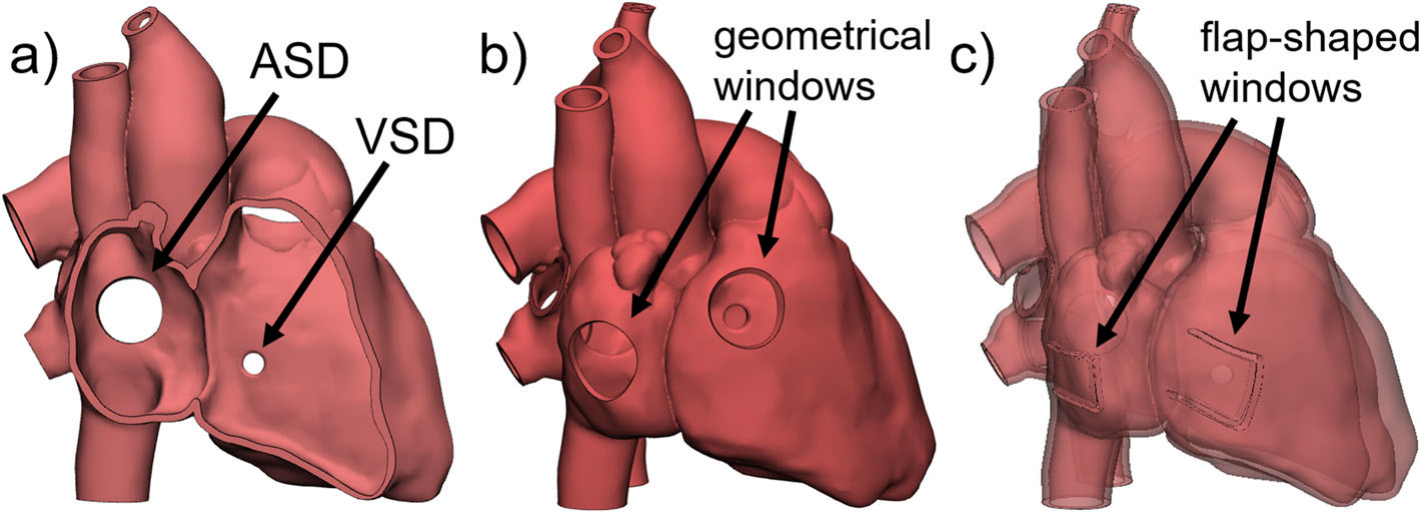
Different methods to create openings and windows for hollow 3D models. The vessels of all depicted 3D hearts have been opened by trimming. **a** Cutting off larger parts and whole structures of a 3D heart allows clear visualization of intracardial anatomical structures, in this case the septum and septal defects (atrial septal defect (ASD), ventricular septal defect (VSD)). **b** Geometric, e.g., circular, windows created by Boolean subtraction of geometric 3D shapes allow examination of the inside of the model, but at the same time maintain a closed shape of the heart. **c** Windows designed as flaps based on curves drawn onto the model surface are particularly appropriate for models printed in flexible material which allows to bend the flap aside. The appearance of heart models with flap-shaped windows is even more closed on the outside

To examine the interior structures of a hollow 3D printed model, it is important to include openings in the model design. Therefore, trimming is one option to cut open vessel endings, or to remove parts or whole structures of the 3D heart. By selecting an area and cutting orthogonal to the screen view all structures lying within this area are deleted. This technique is especially applicable for teaching models, e.g., for anatomy courses, as it allows to create wide openings and clear exposition of anatomical structures inside the heart (Fig. 2a). For models requiring smaller, geometric openings, e.g., catheter training models, windows of defined shape and size are more suitable. Geometric windows in the model wall can be generated by Boolean subtraction of a cylinder or other 3D shape (e.g., cone, cuboid, etc.) (Fig. 2b). Flap-shaped windows might be preferred to give the model a closed appearance on the outside. Printing in a flexible material (AR-G1L, Keyence Corp., Osaka, Japan) processed on a Material Jetting [15] 3D printer (Agilista 3200 W, Keyence Corp., Osaka, Japan) allowed to fold back the flap and to open the view to the inside. One of the approaches to design such a window is to draw a curve defining the window outline onto the surface of the model. The curve can then be used to cut through the model wall and to generate the gap around the window flap (Fig. 2c).

**Fig. 3:**
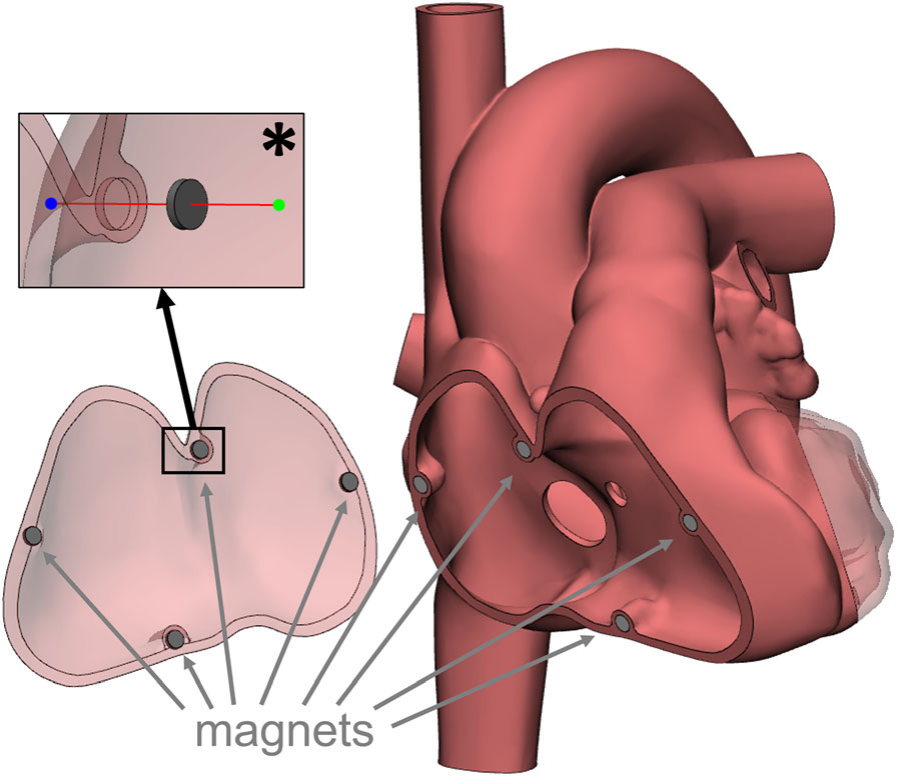
To maintain the complete outer surface of a heart model and allow inspection of interior structures at the same time an assembly model can be created. The individual parts are joined together by including magnets as connectors in the model design. * Detailed view of a magnet, its attachment axis and the corresponding hole at the cutting surface

In cases where at the same time a clear view on the interior structures of the heart as well as a complete representation of the outer shape without openings is desired an assembly model joined together with magnets can be created. Therefore, the 3D heart is cut maintaining all parts. Boolean subtraction of small cylinders in the size of the magnets positioned on opposite cutting surfaces leads to the creation of holes for magnet attachment once the model is printed in 3D (Fig. 3).

### Creation or closure of anatomical defects

**Fig. 4:**
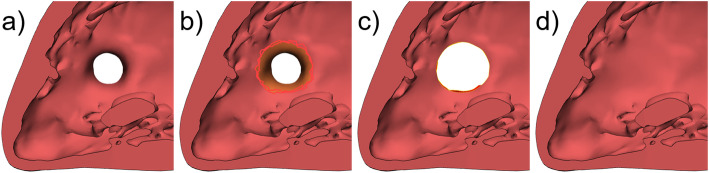
Closure of an anatomic hole in a heart model. **a** View on ventricle septum of 3D model with VSD. **b** Marking of the surface around the VSD (orange). **c** Deletion of the marked area. **d** Closure of the defect and post-processing of the edited area to mask the transition of old to new surface

The creation of a natural anatomical hole (e.g., atrial septal defect (ASD), ventricular septal defect (VSD), patent ductus arteriosus (PDA)) or the closure of such can also be of interest to design heart model variants (Fig. 4a). Engineering operations on digital models allow to mark (Fig. 4b), delete (Fig. 4c), and replace parts of the model’s structure by new surfaces (Fig. 4d). Further post-processing by smoothing and rearranging the triangles of the surface mesh within the treated area makes the design changes invisible.

### Local adaptation of structure size

**Fig. 5:**
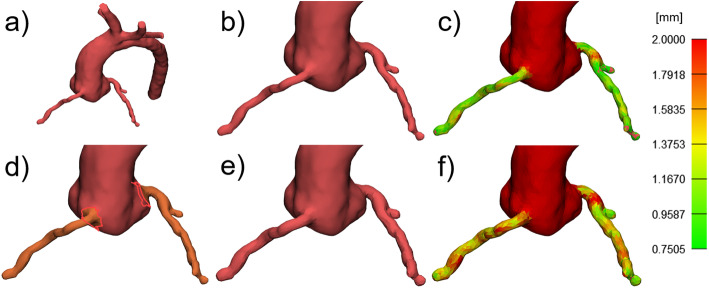
Adaptation of structure size or wall thickness by local offset operations. **a** 3D model of the aorta. **b** Coronaries as segmented from original CT scan. **c **Wall thickness analysis largely showing values < 1mm (critical value chosen for this example) at the coronaries. **d** Marking of the coronary surface to define the region for local size adaptation. **e** Application of a local offset operation to shift the marked triangles towards the outside and, thereby, dilate the coronaries. Post-processing of the transition area conceals surface modifications. **f** Renewed analysis shows wall thickness values > 1mm

Anatomical variations of educational and training models can also be created by local adaptation of structure size by applying a local offset to the model surface. The local manipulation of size can be applied to single structures in contrast to scaling the complete heart, e.g., to left or right atrium (LA, RA) or ventricle (LV, RV) as well as selected regions, e.g., parts of a vessel to simulate aneurysms. In these cases, a local offset is applied to the outside for dilation and to the inside for shrinkage. In addition, 3D printing related design rules - depending on the printing technique and material - can require local size adaptation. One of the criteria for a successful print is to comply with the minimum possible wall thickness. Thus, structures of a 3D model which are thinner should be dilated to avoid failure of the print or breakage of the model. The process of creating local changes to the size or thickness of selected structures can be illustrated, for example, on the 3D model of an aorta with coronary arteries (Fig. 5a and b). The original thickness of the concerned structure can be analyzed by wall thickness analysis tools presenting the analysis results by color mapping (Fig. 5c). According to the targeted minimum wall thickness (depending on printing technique and material as well as the application of the model), thinner or smaller structures can be adapted by marking the concerned area (Fig. 5d) and applying a local offset operation to it (Fig. 5e). Post-processing by smoothing and improving the triangle mesh makes the transition between the maintained and the modified surface invisible. A second analysis verifies whether the aimed wall thickness values were reached (Fig. 5f).

### Pathway simulation

**Fig. 6:**
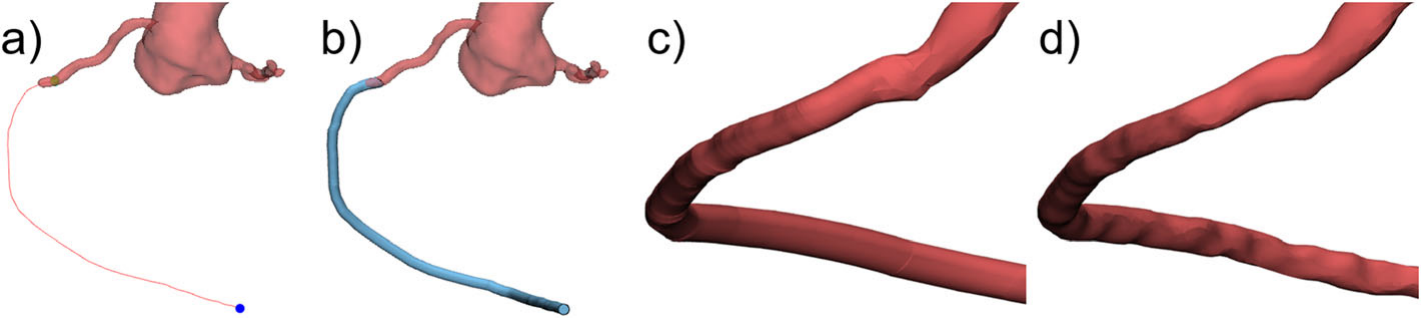
Extension in length and pathway definition for tubular structures. **a** Curve defining the new pathway of a coronary artery. **b** Cylindrical tube created along the new coronary pathway with defined diameter. **c** Generated surface showing a smooth, cylindrical shape letting it appear unnaturally. **d** Design of irregularities into the engineered coronary surface making the anatomical structure of the computed tube look realistic

For tubular structures such as long or peripheral vessels the extension in length or change of pathway can be relevant, e.g., in 3D training models for catheterization. This anatomical modification requires definition of the new (longer and/or different) pathway by an engineered curve (Fig. 6a) and of its outer diameter (Fig. 6b). The resulting tubular structure has a smooth cylindrical shape which does not appear realistic or natural (Fig. 6c). Irregularities can be included into the surface using local push-and-pull operations. This will make the geometric shape appear more natural, like a real human anatomical structure (Fig. 6d).

### Modeling of cardiac muscular structures

**Fig. 7:**
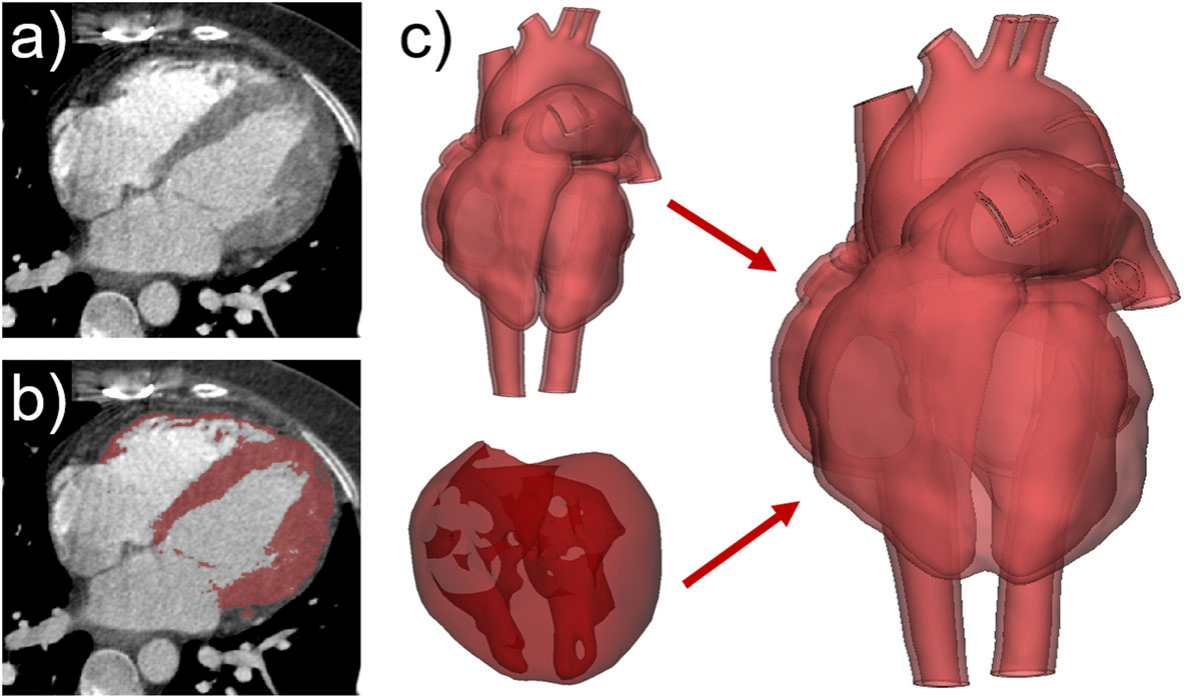
Boolean operations as essential part of the design process of 3D heart models including the myocardium. **a** Heart muscle visible on axial CT scan. **b **Segmentation of the heart muscle (red) performed by local thresholding techniques. **c** Combination of the hollow blood volume and the myocardium by a series of Boolean operations maintaining the correct anatomical intracardiac structure of the ventricles as well as the smooth outer surface of the myocardium

Another powerful set of tools to perform engineering on anatomy are Boolean operations. They are, for example, required to create heart models representing not only a hollowed version of the blood volume but also the muscle, i.e., the myocardium of the heart. Segmentation of the blood volume visible in a CT or MRI dataset results in 3D heart models representing the intravascular and intracardiac structures of a patient. As hollowing for 3D printing is performed by adding an artificial wall of uniform thickness to the outside of the blood volume the muscular structures of the myocardium and intraventricular septum are not represented in the model. However, the myocardium can be visualized in CT and MRI scans (Fig. 7a). Hence, it can be segmented and transformed into an STL of the original, physiological anatomy of the heart muscle (Fig. 7b). A closed and smooth muscle-like outer surface is achieved by smoothing and applying gap-closing tools to the 3D model of the myocardium. These operations will also change the inner surface, i.e., the structure of the ventricles. For correct representation of the heart muscle and ventricular septum as well as the intracardiac surface, the myocardium and the hollow model of the blood volume must be combined. Boolean union of the myocardium and the outer surface of the hollow blood pool results in the outer surface of the final heart model. Subsequent Boolean subtraction of the inner surface of the hollow blood volume from the generated computational mesh will re-establish the correct intracardiac anatomy of the 3D heart model with myocardium (Fig. 7c).

## Methods in applied cases

The presented techniques for the creation of non-patient-specific (= not showing the same anatomy as the original CT or MRI) variants of 3D hearts have been utilized to create educational models for anatomy courses as well as training models for hands-on catheter workshops in pediatric cardiology. Four examples of engineered 3D CHD models are presented in this study.

### Design of perimembranous and muscular VSD (pmVSD, mVSD) on a heart model with PDA

**Fig. 8:**
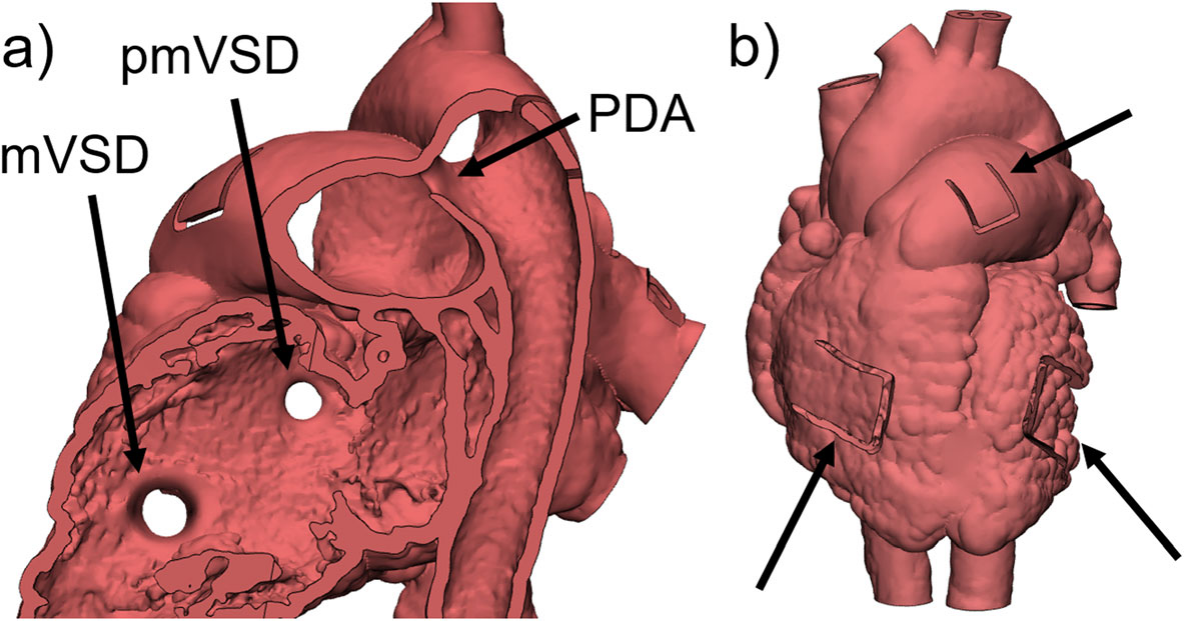
Design of pmVSD and mVSD on a heart model with PDA. **a** The outlines of the VSDs were drawn on the surface of both sides of the ventricle septum and connected by a tubular surface. The resulting cone-shaped object was subtracted from the original model via Boolean operations. **b** Flap-shaped windows (marked by arrows) were included to enable examination of the interior of the model once 3D printed in flexible material

Initially, a hollow model was generated from the contrast-enhanced CT dataset of a pediatric patient with PDA. A pmVSD and mVSD were then artificially created in the ventricle septum. To mimic the correct anatomical appearance of a VSD, a hole with larger opening towards the LV was created. In a first step, circular curves were drawn on each side of the septum representing the larger inlet and smaller outlet of the VSD. By designing a larger opening towards the LV, hemodynamic circumstances, i.e., higher pressure values in the LV compared to the RV, were taken into account. Connection of the curves with a tubular surface resulted in a negative of the septal defect. The 3D object was then subtracted from the original heart model by Boolean operations. The procedure was equally performed for the pmVSD and the mVSD respectively (Fig. 8a). As the model was intended to be used for teaching purposes, flap-shaped windows were included in the design and it was printed in flexible material (Fig. 8b).

### Series of heart models with ASD showing the long-term hemodynamic effect of the CHD on the right atrial and ventricular wall

**Fig. 9:**
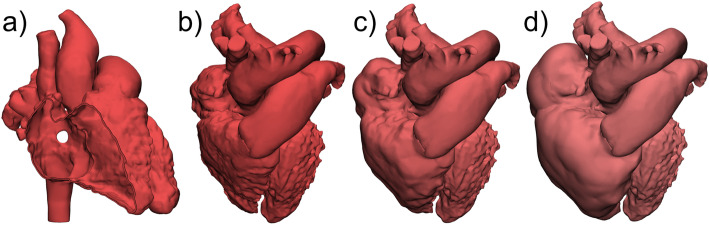
Series of heart models with ASD showing the long-term hemodynamic effect of the CHD on the right atrial and ventricular wall. **a** An ASD was designed on a 3D model based on the dataset of a patient with PDA. **b** Stage 1: No changes to RA and RV. **c** Stage 2: Dilated RA and RV showing the effect of increased mechanical load on the cardiac structures. **d** Stage 3: Further dilation of RA and RV illustrating the severe long-term effect of an untreated ASD

A series of teaching models has been designed to illustrate the possible hemodynamic impact of an ASD to the right atrial and ventricular wall. The malfunction of the septum leads to a left-to-right shunt across the ASD according to the physiological pressure gradient. While the blood volume in the right heart increases, the right heart structures cannot withstand the additional mechanical load in the long term. Thus, in case the ASD remains untreated the RA and RV will dilate over time [16]. The same dataset as described in the first example was used for the ASD models closing the PDA and implementing an ASD (Fig. 9a). The first model of the series represented a heart not suffering from the hemodynamic effects of the ASD for a long time. Thus, the right atrial and ventricular wall were not changed. The widest point between septum and right atrial as well as ventricular wall were about 30mm for this model (Fig. 9b). For the second model the RA and RV were dilated by around 10mm in orthogonal direction to the anterior surface of the structures to show the beginning enlargement due to increased mechanical load (Fig. 9c). Further dilation of the right heart structures by another 10mm was performed to design a third stage of the 3D heart with ASD (Fig. 9d).

### Implementation of simplified heart valves and addition of the myocardium to a right heart model with pulmonary valve stenosis (PS)

**Fig. 10:**
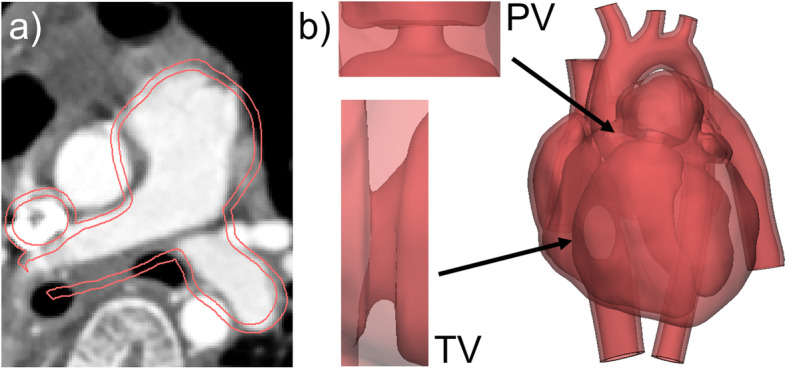
Implementation of simplified heart valves and addition of the myocardium to a right heart model with PS. **a** Contours (red) of the 3D model of the right heart visualized on the axial view of the original CT scan. The pulmonary valve (PV) was given a bulky shape to simulate the morphological changes to the PA caused by the PS. **b** For hands-on catheter trainings the model was modified by including a simplified pulmonary and tricuspid valve (TV), the structures of the left heart as well as the myocardium

The original dataset represented an adult heart with narrowing of the left pulmonary artery (PA) from which a 3D model of the right heart structures was created. The 3D heart was then adapted to the requirements determined by its use as a training model in hands-on catheterization workshops, specifically to practice balloon dilatation interventions on patients suffering from PS. First, the narrowing of the PA was digitally dilated by engineering operations such as the application of a local offset. The pulmonary valve (PV) was given a bulky shape to mimic the morphological transformation caused by the stenosis over time (Fig. 10a, red contour of the 3D model visualized on an axial CT scan of the original dataset). Additionally, “walls” with center holes were constructed at the positions of the pulmonary and tricuspid valve (TV). Shape of the wall and holes were designed and modified based on the input and feedback of a proficient pediatric cardiologist. The goal was to achieve a realistic catheter pathway for PS interventions and a haptically - through the catheter - noticeable representation of the narrowing caused by the valves. The intraventricular structures were smoothed extensively to make catheter manipulation inside the heart model easier and to avoid the tip of the catheter to get stuck in trabecular structures. The left heart structures as well as the myocardium, both derived from the original CT scan were then combined with the right heart model with PS by Boolean operations (Fig. 10b). The finalized 3D heart was then scaled by multiplying factors of 0.8 and 0.55 to provide 3 sizes for training courses, i.e., an adult, teenage and infant patient with PS.

### Integration of a constructed 3D model of the aortic valve (AV) into a pulsatile left heart model with coarctation of the aorta (CoA)

A 3D model has been designed specifically for advanced hands-on catheter trainings on an artificial beating heart. For the training, the model is connected to a closed circulation system filled with water and stimulated with pulsatile flow. The pulsatility is induced by a ventricular assist device (VAD; EXCOR VAD P25P 25ml, Berlin Heart GmbH, Berlin, Germany). It simulates the patient’s heartbeat in the flexible 3D printed heart model allowing further training applications for the catheterization simulation setup such as intracardiac pressure measurements and angiographic imaging using contrast agent.

**Fig. 11:**
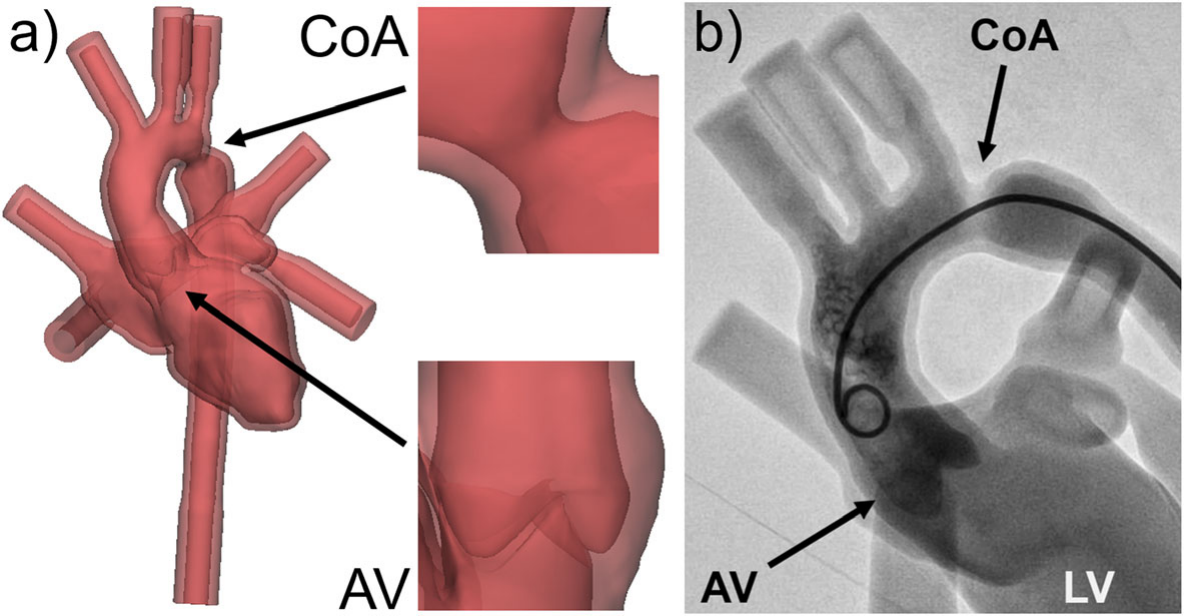
Integration of a constructed 3D model of the aortic valve (AV) into a pulsatile left heart model with CoA. **a** To create a pulsatile training setup for CoA balloon dilatation, design changes were applied to a left heart model. The CoA was created and enforced, an engineered AV was implemented, and vessel endings were closed or adapted (descending aorta, inferior right pulmonary vein) for connection to a closed circulation system with pulsatile flow. **b** The water-filled, beating 3D heart model allows advanced catheter training including angiographic imaging with contrast agent (left ventricle (LV))

The original CT dataset was the same as for the model with PS described before. For the pulsatile 3D model an STL was generated from the left heart structures and the aorta as visible in the scan. A stenosis was then designed at the aortic isthmus for hands-on training of CoA balloon dilatation. Due to intended repetition of the exercise the wall at the CoA was locally enforced to increase resistance and durability. The intracardiac surface was again smoothed extensively to facilitate catheter manipulation during training. To keep the water inside the pulsatile circulation system the endings of the heart vessels were closed off. Geometric tubes compatible with the connectors of the flow system were designed at the endings of the descending aorta and the inferior right pulmonary vein. They remained open for direct connection to the pulsatile system by tube connectors. An engineered STL of an AV was implemented instead of the simplified “wall with hole” initially used for the PS model (Fig. 11a). This design step made the model look even more realistic under angiographic imaging when contrast agent was induced (Fig. 11b).

## Discussion

It has been demonstrated that a range of variant CHD models can be created by modifying existing patient-specific 3D heart models instead of starting with and relying on CT or MRI data. This is specifically beneficial for 3D printing of pediatric cases where minimization of radiation (CT) and sedation (MRI) is key. For 3D printing of the heart valves, 3D ultrasound images have been used [17, 18]. However, the technique is not (yet) applicable for 3D reconstruction of the entire heart. The methodologies presented in this study provide an alternative solution to create a 3D heart model with the desired pathology despite the lack of CT, MRI, or 3D ultrasound data.

The 3D hearts modified by engineering operations on the original anatomy presented in this study have successfully been utilized as educational models (PDA model with pmVSD and mVSD, ASD series). Several research groups have investigated and confirmed the advantages of utilizing 3D printed heart models for education of medical students [19, 20], medical personnel [21–23], as well as patients and their families [24–26]. For educational models it has been shown in this report that several pathologies can be included in one 3D printed heart. This approach saves printing material and costs. Moreover, it has been demonstrated that local changes can be applied to the 3D anatomy to modify selected heart structures. A series of ASD models illustrated the long-term effects of non-treatment, i.e., the change of the affected cardiac structures at different stages over time. A limiting factor of the presented approach is that it can lead to reduced definition in the treated areas, e.g., in the presented model the RV trabeculation. This is specifically the case for large dilation factors (in this case around 30 and 60 % of the original cavity size). The effect can be neglected, if the loss in accuracy does not affect the training purpose of the model. The idea can also be transferred to other CHD cases to show the consequences of advanced, untreated diseases at stages of which datasets might not even exist. Meyer-Szary et al. [27] have 3D printed the model of a patient with aortic aneurysm and aortic dissection resulting from untreated CoA. The model was based on the patient’s CT scan and its intended use was for training and education. With our approach of changing the morphology of existing 3D models the creation of teaching models is not restricted by the incidental availability of the data of such patients. In addition to anatomy related modification, local adaptation of computational meshes also helps to ensure printability considering rules for 3D printing, e.g., regarding the wall thickness.

The designed and modified 3D hearts have also been used as hands-on catheter training models for pediatric cardiologists (PS model, pulsatile CoA model). Nguyen et al. [28] have published the use of 3D printed cardiac models in a similar training setup for adult cardiologists. It has been shown in this study, how changing the original morphology of 3D hearts can help to adapt the complexity to the training goals of a workshop and the experience level of the participants. In this regard, a simple approach is scaling, as the size of the heart – natural or 3D printed - affects the degree of difficulty in the performance of catheterization. Thus, trainees can start practicing a procedure on an adult size heart and slowly migrate to smaller versions with increasing requirements to their dexterity but concentrating on one disease and procedure. It must be considered that simply scaling a heart model in size might not reflect different age-range depictions of morphology as different structures grow at different rates. To overcome this limitation in creating a realistic 3D heart discrepancies between scaled model structure and expected age-related morphology should be managed by further modifications on the structure of the scaled model. Further methods shown in this study related to digital engineering workflows allowing representation of the heart valves and the cardiac muscular structures. These are not included in a 3D heart model when following the usual workflow, i.e., segmentation of the blood pool in a CT or MRI scan and hollowing the blood volume. The presented engineering techniques helped to successfully integrate the myocardium and ventricular septum as well as a constructed AV with 3 leaflets. This resulted in a more realistic representation of the training models under X-ray imaging during catheterization training.

With the methodologies presented in this study, almost any changes can be applied to the shape and structure of a computational mesh using image processing and computer aided 3D design operations. However, there is an obvious risk of creating unrealistic anatomical findings when applying digital design and modification techniques, especially for engineers lacking medical education. The accuracy of 3D modeling and 3D printing, i.e., the dimensionally correct and complete representation of a patient’s anatomy reconstructed from a CT or MRI in the 3D model, has been investigated thoroughly [29–36]. The lack of a clinical dataset for comparison of the resulting model can potentially be a limiting factor of designing and modifying medical 3D models. For this reason, whenever designing a non-patient-specific 3D model it is crucial for the engineer not to get out of touch with reality. It can be helpful to incorporate the information from additional data, e.g., ultrasound imaging or catheterization video sequences to verify design changes. In the end though, a close collaboration between medical engineers and experienced cardiologists or cardiac surgeons is indispensable for the creation of realistic non-patient-specific anatomical 3D heart models. Taking this advice into account, the described boundlessness of the introduced engineering techniques provides the possibility of creating 3D heart models resembling pediatric patients with no indication for CT or MRI imaging, e.g. most cases of hypoplastic left heart syndrome prior to Stage I Norwood operation [37]. For very complex CHD cases, e.g., transposition of the great arteries prior to arterial switch, it can be helpful to start with a 3D model generated from a CT or MRI scan with at least similar pathology to facilitate the engineering process.

The demonstrated engineering techniques were applied to create educational and training models of CHD cases. The design of more complex cases such as the afore mentioned transposition of the great arteries prior to arterial switch for which CT or MRI imaging are not indicated is planned. To pursue the objective of creating realistic 3D models, we want to further explore the possibilities of 3D ultrasound segmentation and combination of imaging modalities for real-life representation of all four heart valves in the 3D heart models as described by Gomez et al. [17]. Our digitally engineered heart models have been utilized as educational models as well as hands-on catheter training models for pediatric cardiologists, so far. Yoo et al. [6, 38–43] and also other research groups [44–47] reported about their positive experience in using 3D hearts based on CT or MRI scans in hands-on training courses for pediatric cardiac surgeons. Therefore, it is planned to evaluate the applicability of digitally designed non-patient-specific 3D hearts for hands-on training in pediatric cardiac surgery.

## Conclusions

The investigations of this study show that it is possible to virtually adapt a patient-specific 3D heart model generated from a CT or MRI scan to create different non-patient-specific anatomical variants and CHD cases, all based on one single patient dataset. The examples of non-patient-specific 3D printed heart models presented in this study were used as educational models as well as for hands-on trainings in diagnostic and interventional pediatric cardiology. 3D models can be simplified or amplified in complexity to adapt their structure to training levels and goals. Even pathologies with no indication for CT or MRI imaging can be constructed and 3D printed. The presented approach was applied to education and training in pediatric cardiology but can potentially be transferred to other fields such as pediatric cardiac surgery. Expectedly, all medical fields can benefit from simulation training using engineered non-patient-specific 3D printed models.

## Data Availability

The datasets generated during the current study are not publicly available but are available from the corresponding author on reasonable request.

## Abbreviations

ASD: Atrial septal defect
AV: Aortic valve
CHD: Congenital heart disease
CoA: Coarctation of the aorta
CT: Computed tomography
LA: Left atrium
LV: Left ventricle
MRI: Magnetic resonance imaging
mVSD: Muscular ventricular septal defect
PA: Pulmonary artery
PDA: Patent ductus arteriosus
pmVSD: Perimembranous ventricular septal defect
PS: Pulmonary valve stenosis
PV: Pulmonary valve
RA: Right atrium
RV: Right ventricle
TV: Tricuspid valve
VAD: Ventricular assist device
VSD: Ventricular septal defect
